# Supplementary material for: Strategy for Fast Decision on Material System Suitability for Continuous Crystallization Inside a Slug Flow Crystallizer
Source: Micromachines (Basel). 2022 Oct 21;13(10):1795. doi: 10.3390/mi13101795 (PMC9610778; doi:10.3390/mi13101795)
Supplement: Supplementary file 1 [file micromachines-13-01795-s001.zip › micromachines-1974269 - Supplementary Information.pdf]

micromachines

Supporting Information

Strategy for Fast Decision on Material System Suitability for Continuous Crystallization Inside a Slug Flow Crystallizer

Anne Cathrine Kufner [1,†], Adrian Krummnow [2,3,†], Andreas Danzer [2], and Kerstin Wohlgemuth [1,*]

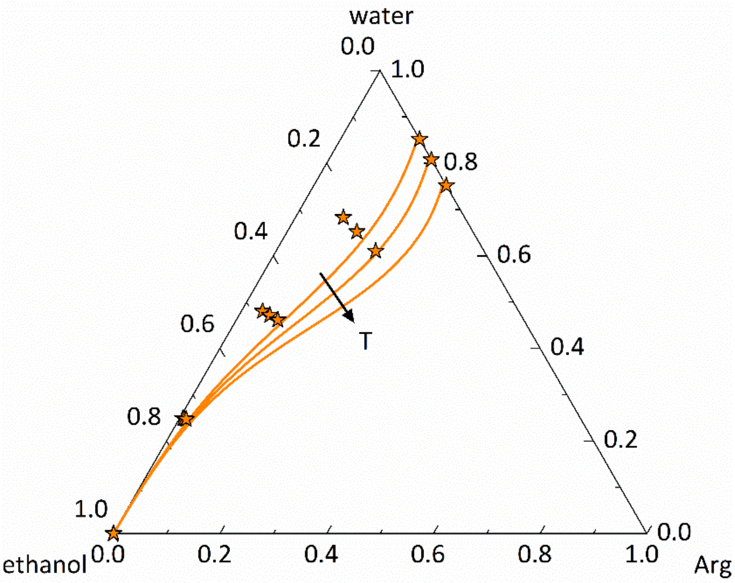

Figure S1. Ternary phase diagram of Arg/water/ethanol at 0.1 MPa with compositions given in mass fractions: Solubility lines were predicted in this work using PC-SAFT, and symbols denote solubility measurements performed in this work. The arrow indicates the direction of increasing temperature from 20 °C to 30 °C, and 40 °C.

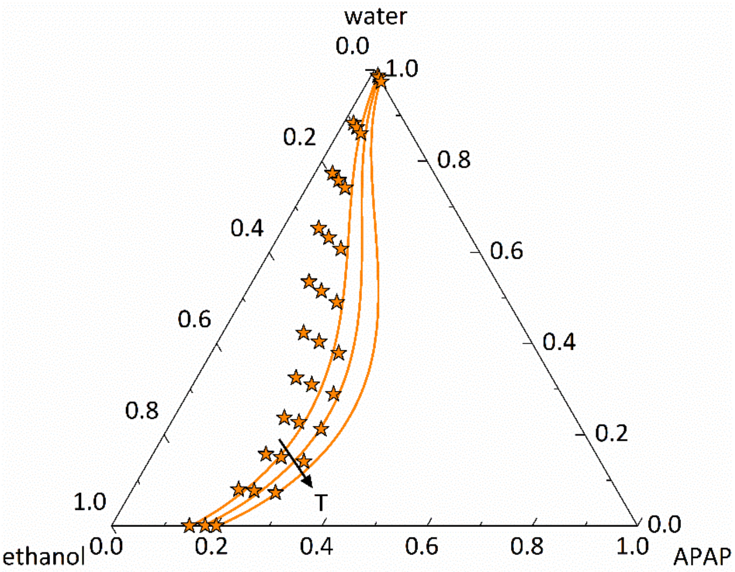

Figure S2. Ternary phase diagram of APAP/water/ethanol at 0.1 MPa with compositions given in mass fractions: Solubility lines were predicted in this work using PC-SAFT, and symbols denote solubility measurements from JIMENEZ AND MARTINEZ [62]. The arrow indicates the direction of increasing temperature from 20 °C to 30 °C, and 40 °C.

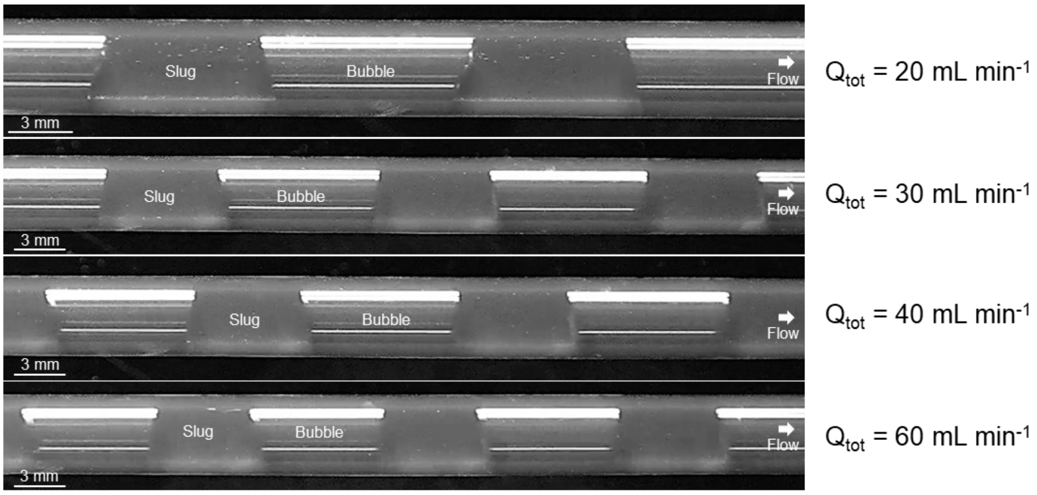

Figure S3. Images of water slugs (w_{EtOH} = 0 %) at the end of tubing (L = 7.5 m) at different total volume flow rates Q_{tot}. The experiments were conducted at ambient temperature ($\vartheta_{amb} \approx$ 22 °C).

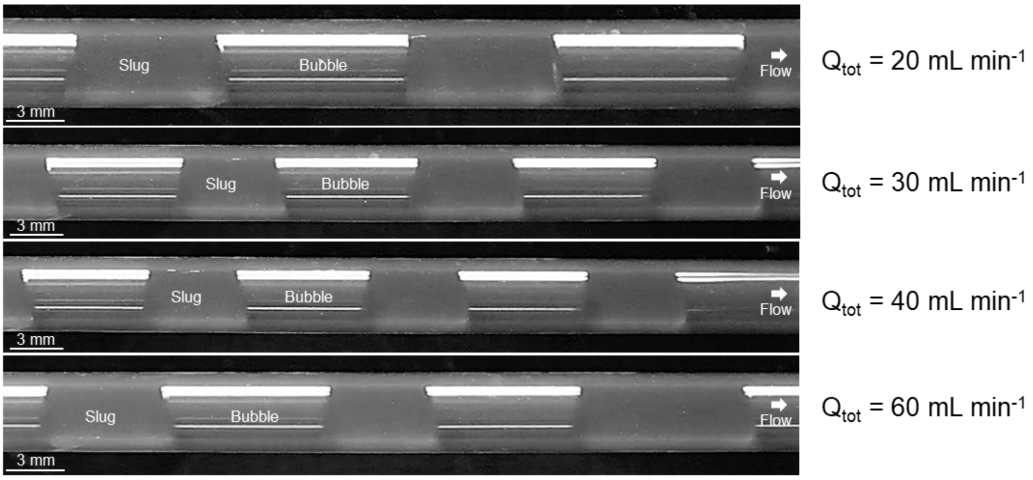

Figure S4. Images of slugs (w_{EtOH} = 10 %) at the end of tubing (L = 7.5 m) at different total volume flow rates Q_{tot}. The experiments were conducted at ambient temperature ($\vartheta_{amb} \approx$ 22 °C).

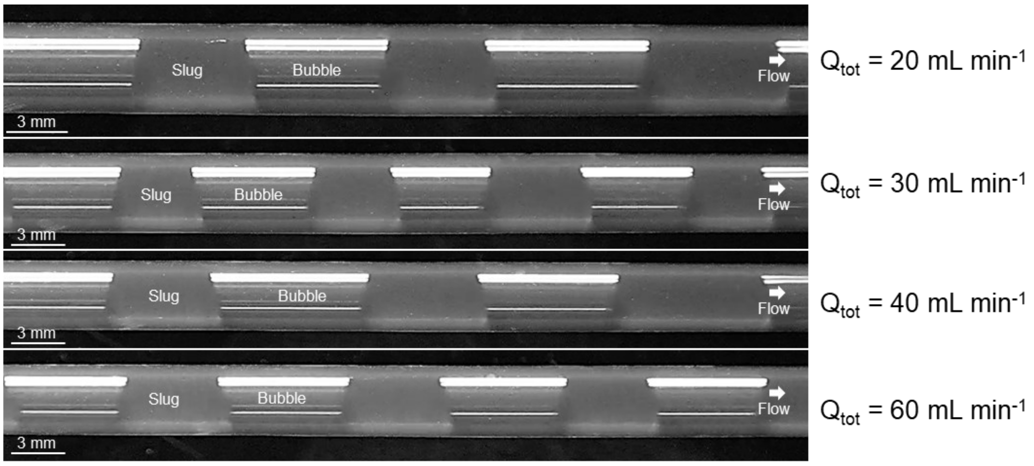

Figure S5. Images of slugs (w_{EtOH} = 20 %) at the end of tubing (L = 7.5 m) at different total volume flow rates Q_{tot}. The experiments were conducted at ambient temperature ($\vartheta_{amb} \approx$ 22 °C).

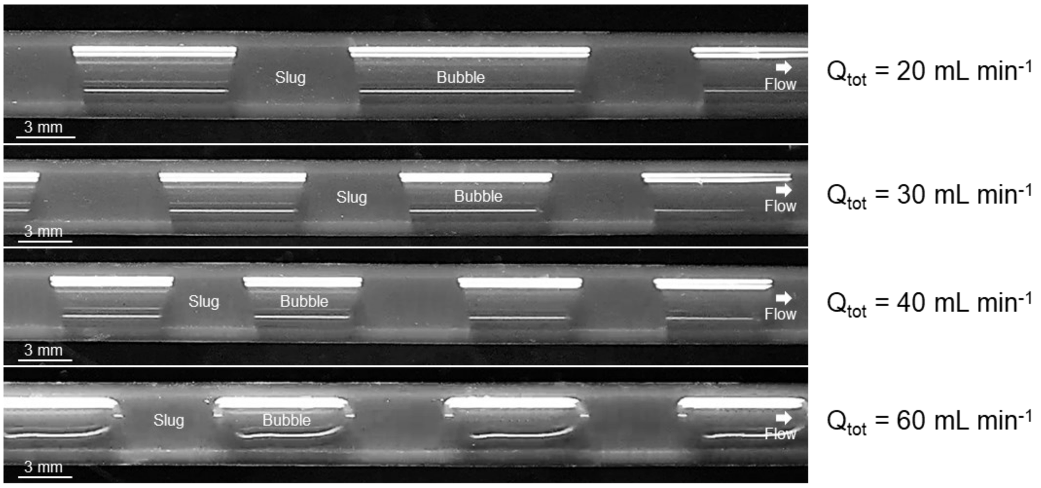

Figure S6. Images of slugs (w_{EtOH} = 30 %) at the end of tubing (L = 7.5 m) at different total volume flow rates Q_{tot}. The experiments were conducted at ambient temperature (ϑ_{amb} ≈ 22 °C).

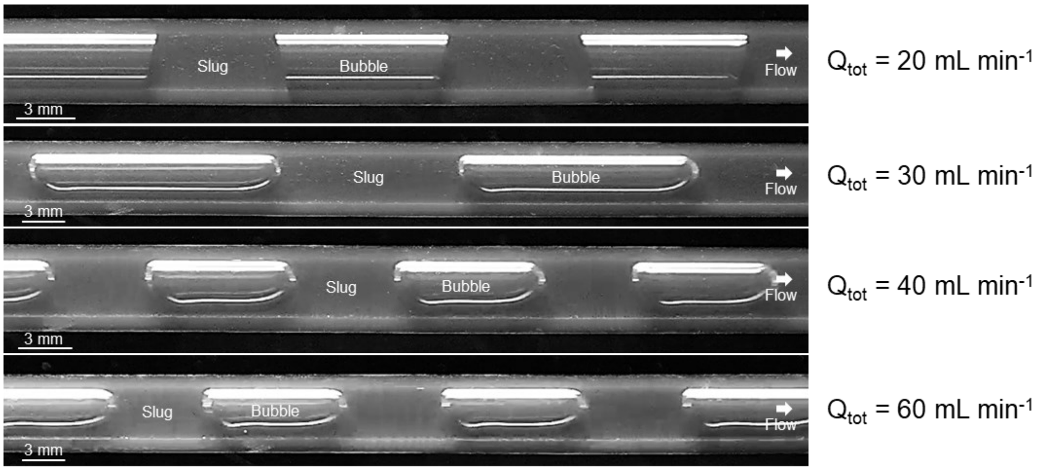

Figure S7. Images of slugs (w_{EtOH} = 50 %) at the end of tubing (L = 7.5 m) at different total volume flow rates Q_{tot}. The experiments were conducted at ambient temperature (ϑ_{amb} ≈ 22 °C).
